# Developmental dynamics of transcription initiation highlight promoter shifts as a multifaceted regulatory mechanism in barley

**DOI:** 10.3389/fpls.2026.1975716

**Published:** 2026-10-29

**Authors:** Simon Pavlu, Ondrej Kopecky, Pavla Navratilova, Hana Simkova

**Affiliations:** 1 Centre of Plant Structural and Functional Genomics, Institute of Experimental Botany of the Czech Academy of Sciences, Olomouc, Czechia; 2 Department of Cell Biology and Genetics, Faculty of Science, Palacky University, Olomouc, Czechia

**Keywords:** BRI1, *cis*-regulatory element, *Hordeum vulgare*, SUS1, terpene cyclase, TOP motif, uORF

## Abstract

Alternative promoter usage is a widespread regulatory mechanism whereby a single gene utilizes two or more transcription start sites (TSSs), enabling the production of functionally distinct transcript and protein isoforms in a tissue-, developmental stage-, or condition-specific manner. While this phenomenon has been extensively studied in metazoans, genome-wide characterization of alternative promoter usage in crop species is still lacking. Here, we use Cap Analysis of Gene Expression (CAGE) to characterize genome-wide TSS dynamics across three stages of barley embryo development and the developing inflorescence. We identify 2,129 developmental promoter shifts relating to 1,353 genes, corresponding to ~5% of genes expressed in at least one of the four developmental stages. One-third of these shifts coincided with significant differential gene expression, and the affected genes were enriched for developmental and regulatory functions, particularly those related to cell wall biosynthesis and hormone signaling. Furthermore, for 521 genes, we predict potential effects of alternative TSS usage on translational efficiency or protein structure, suggesting regulatory consequences that extend beyond changes in transcript abundance. Together, our findings suggest that alternative promoter selection is an important regulatory mechanism during barley development and provide new insights into the developmental regulation of gene expression in cereals. Moreover, we provide a gene-level catalog of developmental promoter shifts in barley, offering a valuable resource for promoter editing and engineering to fine-tune gene expression for desirable agronomic traits.

## 1 Introduction

Transcription initiation is a fundamental and precisely regulated step in gene expression. Many genes can initiate transcription at multiple genomic locations, often in response to developmental or environmental signals. Alternative transcription start sites (TSSs) can occur within a single promoter or involve switches between separate promoter regions; the distinction is primarily determined by the distance between the TSSs, which can range from a few nucleotides, implying shared core promoter architecture, to several kilobases, enabling exposure to distinct regulatory environments (Alfonso-Gonzalez and Hilgers, 2024). Knowledge of alternative promoters and an understanding of their usage across an organism’s life cycle and in response to environmental stimuli provide a valuable foundation for promoter engineering and synthetic biology approaches aimed at fine-tuning gene expression in a tissue- or condition-specific manner.

Alternative promoter usage (APU) has been studied most extensively in metazoans, where promoter switching modulates gene expression across developmental and physiological contexts (Davuluri et al., 2008; Nepal et al., 2013; Tan and Sun, 2025), accompanies major developmental transitions, including the maternal-to-zygotic switch (Haberle et al., 2014), and has been implicated in pathological states such as heart failure and cancer (Gacita et al., 2020; Weber et al., 2023). In plants, alternative transcription initiation has been examined in detail in Arabidopsis, primarily in response to specific environmental and nutritional cues such as light and phosphate availability (Ushijima et al., 2017; Kurihara et al., 2018; Zhang et al., 2022; Reis et al., 2024). Genome-wide insight into developmental promoter dynamics in crop species is therefore still lacking.

APU can influence gene expression at several levels (**Figure 1A**). First, switching to an alternative promoter reshapes the *cis*-regulatory landscape of the transcriptional unit, thereby altering tissue, developmental-stage or condition specificity of gene expression, as well as transcript abundance (Haberle et al., 2014; Schmitz et al., 2022). Second, promoter switching can alter the composition of the 5’ untranslated region (UTR), which in turn influences translational efficiency or mRNA stability through regulatory elements such as the 5’ terminal oligopyrimidine (TOP) motif or upstream open reading frames (uORFs) in mRNAs (Kurihara et al., 2018; Philippe et al., 2020; Zhang et al., 2020). 5’TOP-containing transcripts are regulated through the Target of Rapamycin (TOR) kinase pathway, which links the translation outcome to nutrient availability (Liu and Sabatini, 2020). In vertebrates, regulation via this pathway predominantly affects genes involved in ribosome biogenesis and protein synthesis, whereas in plants, the effect is also extended to other conserved genes that participate in a number of biological processes (Scarpin et al., 2020). The uORFs, in turn, can suppress translation of the main coding sequence by interfering with ribosome scanning, dissociation, stalling, and translation re-initiation. They can also reduce mRNA stability in a context-dependent manner (Wu et al., 2024). Third, alternative transcription initiation within the coding region may disrupt the reading frame and introduce premature termination codons, generating non-productive transcript isoforms, targeted by nonsense-mediated decay (Popp and Maquat, 2013). However, when the reading frame is preserved, the alternative initiation changes the N-terminal coding sequence and associated targeting signals, leading to changes in subcellular localization of the protein or composition of its functional domains (Davuluri et al., 2008; Ushijima et al., 2017).

**Figure 1:**
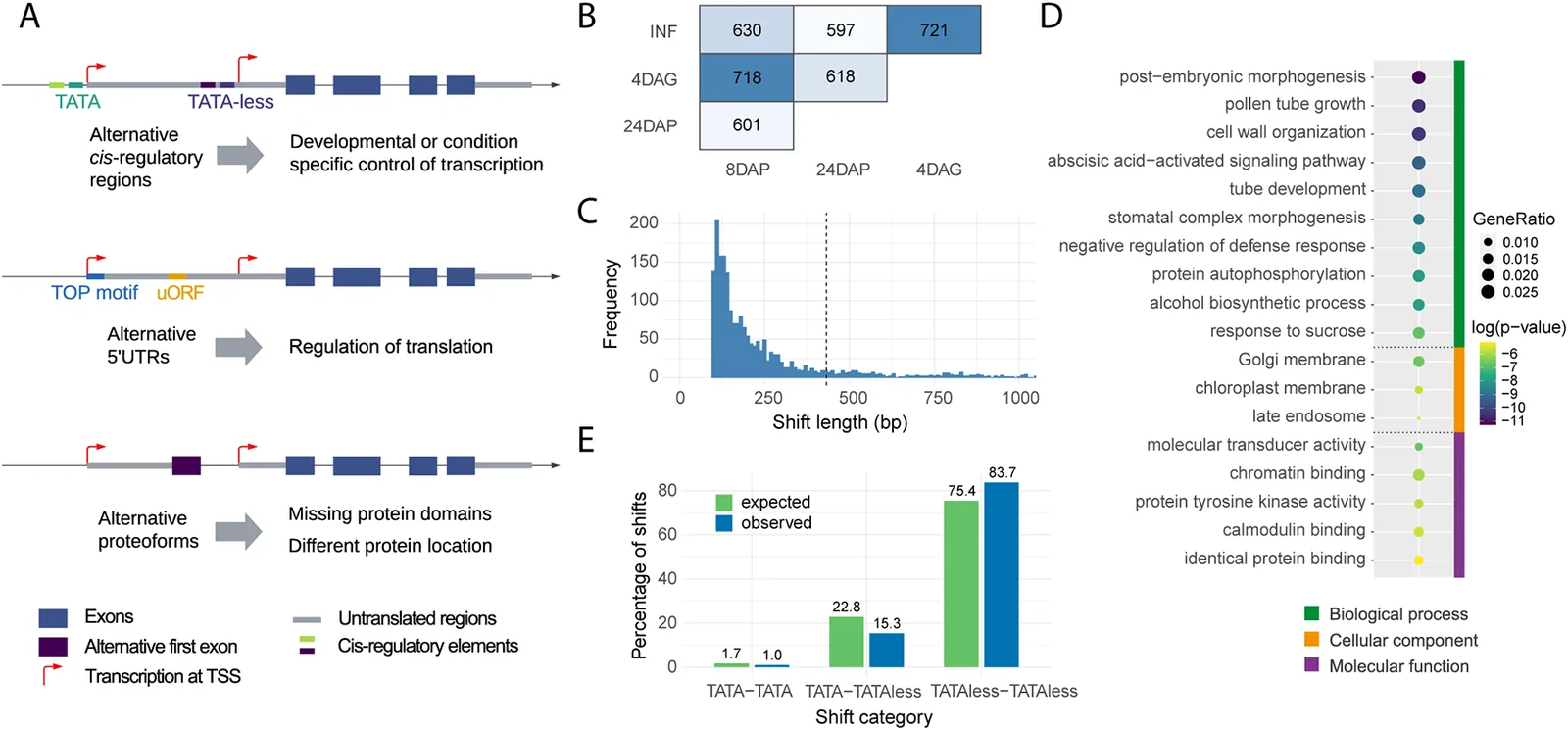
Description of developmental promoter shifts in barley. **(A)** A schematic overview of alternative TSS usage and possible impacts on gene expression. **(B)** The abundance of long-distance (≥ 100bp) promoter shifts across four barley developmental stages, including developing embryo (8DAP, 24DAP), germinating embryo (4DAG) and early inflorescence (INF). The heatmap shows the number of shifts for each pair of stages. **(C)** Distribution of promoter shift distances for shifts ≥100 bp. The mean distance of 432 bp is marked with a dashed line. **(D)** GO term enrichment analysis for 1,353 genes with developmental promoter shifts against the background of all genes expressed in the analyzed stages. The color indicates the significance of the enrichment. **(E)** Expected versus observed frequency of the TATA-box in shifting promoters. Bars show the proportion of shifts classified as TATA–TATA, TATA–TATAless, and TATAless–TATAless, comparing the expected proportions calculated from the frequency of TATA and TATA-less promoters in the whole CAGE promoter dataset (green) versus the observed proportions in the shifting promoter set (blue).

Dissecting any of these mechanisms requires exact knowledge of TSS positions. RNA-seq profiles transcript abundance genome-wide but cannot reliably resolve closely spaced TSSs. In contrast, Cap Analysis of Gene Expression (CAGE) maps TSSs at single-nucleotide resolution by sequencing 5’ends of capped RNAs (Shiraki et al., 2003) and has become a standard approach to promoter annotation in large genome projects (Carninci et al., 2005; The ENCODE Project Consortium, 2012; Forrest et al., 2014).

Using CAGE, we previously defined promoters for 21,610 genes and predicted thousands of alternative TSSs across three developmental stages of the barley embryo (Pavlu et al., 2024), without exploring developmental dynamics of the alternative promoter usage. Here, we created an additional CAGE dataset from young barley inflorescence and conducted systematic identification and comprehensive analysis of developmental promoter switches across four stages of barley development. Specifically, we catalog genome‐wide developmental TSS shifts, assess their impact on transcript abundance and architecture, and evaluate their potential to confer translational control. Using three genes with shifting promoters—*Brassinosteroid Insensitive 1* (*HvBRI1*), *Sucrose synthase 1* (*HvSUS1*) and a terpene cyclase gene—as illustrative examples, we discuss potential functional consequences of particular shift events. To our knowledge, this study represents the first genome-wide analysis of developmental promoter shifts in an important crop species and provides a valuable resource for precision engineering of gene expression in barley.

## 2 Methods

### 2.1 CAGE analysis

Barley cv. Morex was grown in growth chambers at 16/8 hrs light cycle, 16 °C day/12 °C night. Fifty inflorescences at the lemma to awn primordia stages (W3.0–W5.5) were dissected under a stereomicroscope, and total RNA was extracted using the Monarch Total RNA Miniprep Kit (NEB) according to the manufacturer’s protocol, in two biological replicates. The CAGE library preparation and Illumina paired-end (2x150 bp) sequencing were conducted by DNAFORM (Yokohama, Japan) using their proprietary protocols. Read mapping of CAGE tags was done using BWA (version 0.7.17) and HISAT2 (version 2.0.5) as described previously (Pavlu et al., 2024). All CAGE data were clustered using CAGEr (Haberle et al., 2015). The code and specifications are available on GitHub.

### 2.2 Identification of promoter shifts and analysis of differential transcription

Determination of dominant TSSs in individual samples, removal of signals associated with exon-intron boundaries, identification of long and short promoter shifts based on CAGE data, and differential analysis were done using a custom code available on GitHub. We excluded shifts where both TSSs were located further than 1000 bp upstream of the nearest annotated TSS, thereby reducing the risk of possible mis-assigning TSSs of a neighboring unannotated gene. The upper threshold for the promoter shift distance was set at 5000 bp. For analysis of short-distance shifts, we used strict filters to exclude the broad initiation noise. We analyzed only shifts of 5–99 bp and applied a threshold of 0.5 tags per million for each of the two alternative TSSs in the developmental stage with the top level of transcription initiation. Additionally, for each stage we calculated what fraction of CAGE tags initiated at the shifting TSS positions. A shift was considered only if the dominant TSS in the pair changed substantially between stages so that it was covered by at least 60% of the tags in one stage and at most 40% in the other.

To evaluate differences in transcription level for pairs of alternative promoters, CAGE tags overlapping the identified promoter clusters were quantified per stage for each long-distance shift. Differential usage of the two promoters between developmental stages was then assessed using DESeq2 (v1.40.1, Love et al., 2014).

### 2.3 Prediction of regulatory features in transcripts from shifting promoters

To identify uORFs that may arise with promoter shifts, we examined regions between alternative TSSs for in-frame start–stop codon pairs corresponding to translated sequences of ≥10 amino acids, using the *findUORFs* function from the ORFik package (Tjeldnes et al., 2021). For identification of TOP motifs in transcripts, we calculated TOPscore values using a published algorithm (Philippe et al., 2020).

### 2.4 GO term enrichment analysis

For the GO term analysis, we used our plant-centered GO annotation previously generated with the GOMAP software (Wimalanathan and Lawrence-Dill, 2021; Pavlu et al., 2024). The enrichment analysis was performed using the *enricher* function from the clusterProfiler package with default settings. The resulting GO terms were processed with REVIGO (Supek et al., 2011) to reduce redundancy. For visualization, we plotted only GO terms with enrichment scores within the upper quartile. To identify the genes most recurrent across the enriched GO terms, we ranked genes by a weighted contribution score that combines the number of GO terms, in which a gene appears, and the magnitude of its expression change (score = log1p(n) × max_abs_log2FC).

### 2.5 Motif enrichment analysis

Identification of TATA-box motifs was done in regions -25 to -40 bp from alternative TSSs by position weight matrix (PWM)-based motif scanning, using the function *searchSeq* from the TFBSTools package and a plant TATA-box PWM obtained from Eukaryotic Promoter Database (Cavin Périer et al., 1998). Relative score threshold of ≥95% was used for analysis of the canonical TATA-box, and ≥80% for W-box. Transcription factor (TF) motif analyses were carried out in regions -50 to -150 bp from a TSS for each of the shifting promoters (SPs) as well as a background set of all CAGE-defined promoters. Known TF binding sites (TFBSs) were identified using genome-wide predictions generated by FIMO (MEME Suite v5.5.7; Grant et al., 2011), using barley-specific motifs from PlantTFDB (Tian et al., 2020). *De novo* motifs were identified by STREME (MEME Suite; Bailey, 2021) and annotated by comparison to barley PlantTFDB motifs using Tomtom (MEME Suite; Gupta et al., 2007) with --thresh 0.1. Selected motifs were subsequently scanned in the promoter-shift and background sets using FIMO. The used code with specifications is available on GitHub.

### 2.6 Statistical analyses

Unless stated otherwise, all statistical analyses were performed in R, and p-values from multiple comparisons were adjusted using the Benjamini–Hochberg method. Deviation of the observed TATA-box shift architectures (TATA–TATA, TATA–TATA-less, TATA-less–TATA-less) from the frequencies expected given the genome-wide proportions of TATA and TATA-less promoters were assessed with a chi-squared test. Differences in log2 fold-change distributions between shift categories were tested with a Wilcoxon signed-rank test against zero. Enrichment of individual sequence motifs and TFBS modules in shifting promoters was calculated against the background set of all active promoters using Fisher’s exact test. Promoter-variant specificity of motifs and modules was assessed with a binomial test. GO term enrichment was based on the hypergeometric test implemented in the *enricher* function of clusterProfiler with default settings.

## 3 Results

### 3.1 Identification and characterization of shifting promoters

We aimed to characterize genome-wide TSS usage and promoter dynamics across developmental stages of barley. In a previous study (Pavlu et al., 2024), we generated CAGE datasets from three stages of barley embryo, eight days after pollination (8DAP), 24 days after pollination (24DAP) and four days after germination (4DAG) (GEO accession GSE227219), and established a foundation of high-resolution promoterome atlas. Here, we generated an additional CAGE dataset from developing inflorescence (INF), thereby expanding the promoter atlas beyond embryo-specific TSSs and enabling the capture of promoters active in reproductive tissues. Building on this data, we performed systematic identification and comprehensive characterization of developmental promoter shifts.

With all CAGE datasets, we conducted principal component analysis of two biological replicates for each developmental stage, which showed tight clustering of the replicates and clear separation of the stages, indicating high reproducibility and distinct stage-specific transcriptional profiles (**Supplementary Figure S1A**). After merging replicates for each sample, we obtained 52,313,604/57,927,548/60,825,231/75,609,473 CAGE tags for the 8DAP/24DAP/4DAG/INF samples, respectively. Clustering neighboring TSSs with CAGEr (Haberle et al., 2015) provided 48,274 consensus tag clusters expressed in at least one sample (**Supplementary Data S1**), which were assigned to 25,150 genes from the MorexV3 annotation (Mascher et al., 2021).

Further analysis revealed developmental promoter shifts of varying lengths, which we categorized as long (≥100 bp) or short (<100 bp) (**Supplementary Table S1**). Because broad promoters frequently exhibit dispersed transcription initiation across nearby start sites, obscuring the short-shift downstream analysis, we focused predominantly on the long-distance shifts (**Supplementary Data S2**). The number of such shifts identified in pairwise developmental stage comparisons ranged from 597 to 721 events (**Figure 1B**), with a total of 3,885 shifts, of which 2,129 constituted a non-redundant set with an average length of 432 bp (**Figure 1C**). These long-distance shifts, relating to 1,353 genes, were further classified according to their expected effect on gene expression: (i) shifts localized to non-coding regions upstream of a gene, possibly altering promoter architecture, 5’UTR structure or *cis*-regulons, and (ii) shifts affecting the coding region, leading to alternative protein isoforms (**Figure 1A**). Of the 2,129 shifts, 1,790 (~84%) fell into the non-coding class, with 1,414 (~66%) occurring within an annotated promoter region (-500/+100 bp relative to the annotated TSS; **Supplementary Figure S1B**). In contrast, only 339 (~16%) shifts affected the coding region.

To explore the biological processes most associated with promoter shifts, we performed a GO term enrichment analysis on the 1,353 genes exhibiting the long-distance shifts against the background of all 25,150 genes expressed in the analyzed stages. The enriched terms highlighted developmental and regulatory processes, including cell wall organization, auxin transport, protein phosphorylation, and responses to environmental and hormonal stimuli (**Figure 1D**). A recurrent gene analysis, which identifies genes most frequently represented across enriched GO terms (**Supplementary Table S2**), highlighted a prominent role of promoter shifts in the regulation of Golgi-mediated biosynthesis of non-cellulosic cell wall polysaccharides, including pectin and xylan, together with cellulose synthesis at the plasma membrane.

### 3.2 Promoter shifts can change promoter architecture

The use of an alternative promoter can be associated with a change in promoter architecture, which can involve a switch between TATA-box and TATA-less promoter types. To explore this systematically, we utilized a PWM representing the canonical plant TATA-box, generated from promoter sequences deposited in the Eukaryotic Promoter Database. We classified shifts by TATA-box status within 25–40 bp upstream of shifting TSSs into three categories: (i) TATA to TATA, (ii) TATA-less to TATA-less, or (iii) TATA to TATA-less. While TATA-box promoters constitute only a minor portion (~20%) of the entire CAGE dataset, the distribution of architectures of the SPs deviated strongly from random expectation (χ² test, p < 2.2e-16). In particular, shifts between two TATA-less promoters were substantially enriched compared with the random expectation calculated from the genome-wide frequencies of TATA and TATA-less promoters (83.7% observed vs. 75.4% expected), whereas shifts involving TATA-box promoters occurred less frequently (**Figure 1E**). Across developmental stages, the proportion of shifts involving TATA-box and more relaxed W-box promoters remained stable, ranging from 7 to 9% and 37 to 39%, respectively (**Supplementary Figure S1C**).

To assess whether short-distance promoter switches might also alter the core regulatory elements, we examined a subset of 344 short-distance shifts ranging from 5 to 99 bp that exhibited sharp TSS signals and pronounced switching between stages (**Supplementary Data S3**). Almost all of these shifts occurred in TATA-less promoter contexts. Only a single canonical TATA-box was found at one of the shifting TSSs, and a minority (23%) of events involved a W-box at either site. This strong bias indicates that short-distance TSS repositioning predominantly takes place within broadly initiating, TATA-less promoters, where precise positioning relative to core motifs such as the TATA-box is not indispensable for promoter activity.

### 3.3 Promoter shifts aid in regulating gene transcription

To assess possible regulatory impact of promoter shifts, we first investigated whether changes in TSS usage are accompanied by changes in gene transcription. We found that approximately one third of all promoter shift events were associated with significant differential expression for pairs of alternative TSSs (|log2FC| ≥ 2, FDR ≤ 0.05; **Figure 2A**), suggesting the role of alternative TSS usage in regulating transcription strength. To uncover downstream biological processes affected by the substantial expression changes associated with promoter shifts, we performed GO term enrichment analysis on the differentially expressed genes with SPs against a background set of all differentially expressed genes (**Figure 2B**). Out of the GO terms enriched in the set of all SP genes (**Figure 1D**), mainly those relating to post-embryonic morphogenesis remained significant when the change in expression level was taken into account (**Figure 2B**), suggesting that among the developmental stages analyzed, APU is mainly applied in the transition to transcriptional programs governing the early development of barley seedlings. This assumption was supported by the GO term enrichment analysis for SP genes with substantial differential expression against the background of all expressed genes that was done for pairs of developmental stages. This analysis points to the relationship of the most enriched GO terms, including cell wall organization, xylem development and apical protein localization, to the 4DAG stage (**Supplementary Figure S2A**).

**Figure 2:**
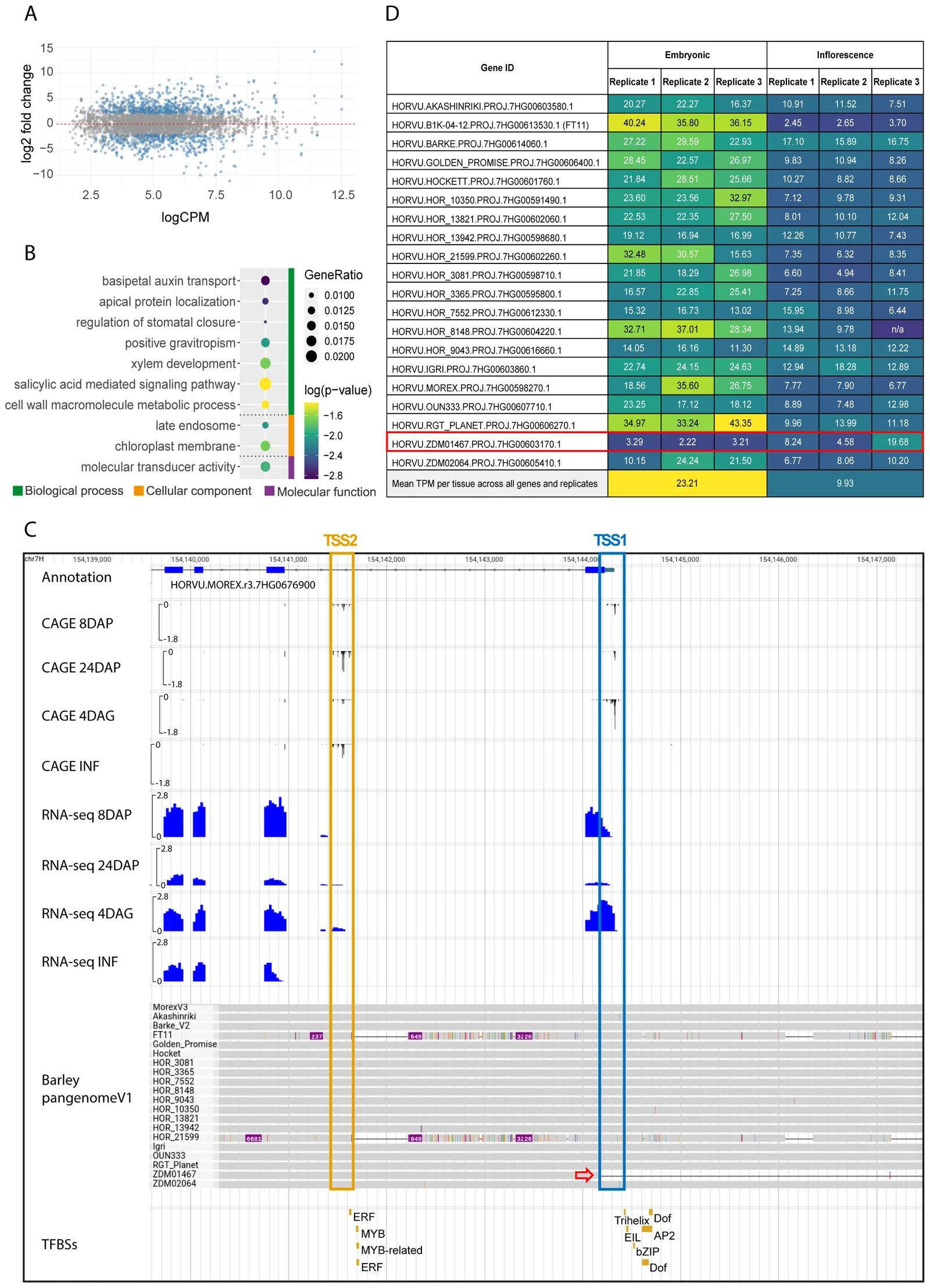
Alternative promoter usage affects gene transcription. **(A)** Differential expression for all TSS pairs undergoing developmental long-distance shifts, based on analysis of CAGE data with DESeq2. The x-axis indicates normalized expression level (logCPM; log counts per million CAGE tags), and the y-axis shows log2-fold change (log2FC) between stages. Shifts associated with significant difference in expression (|log2FC| ≥ 2 and FDR < 0.05) are shown in blue. **(B)** GO term enrichment analysis for the significantly differentially expressed SP genes from **(A)** against the background of all differentially expressed genes in the analyzed samples. The color indicates the significance of the enrichment. **(C)** Promoter shift in gene *HORVU.MOREX.r3.7HG0676900*, encoding a protein from terpene cyclase family. Two alternative TSSs, apparent in both CAGE and RNA-seq data (Kovacik et al., 2024; Mascher et al., 2017), generate distinct transcript isoforms, with the internal TSS (TSS2) skipping the first exon. Further tracks show sequence diversity across genotypes of barley pangenomeV1 (Jayakodi et al., 2020), and TF-family blocks (based on PlantTFDB prediction, p-value < 1e-5) in regions up to 500 bp upstream of each TSS. For details, see BarleyEpiBase. The red arrow points to a deletion in genotype ZDM01467 that encompasses TSS1 and the adjacent promoter proximal region. **(D)** Transcription analysis of *HORVU.MOREX.r3.7HG0676900* (*HORVU.MOREX.PROJ.7HG00598270*) and its orthologs in a barley pantranscriptome study (Guo et al., 2025). Visualization of the data was obtained from PanBARLEX, values are in transcripts per million (TPM). Note that the deletion of the TSS1 region in ZDM01467 is associated with a strongly reduced transcription in the germinating embryo (the Embryonic sample), while expression in the Inflorescence sample remains unaffected, supporting different developmental regulation of the alternative promoters.

We next asked whether different classes of promoter shifts show distinct effects on transcriptional output. Classification of promoter shifts into the two functional categories revealed distinct expression patterns between shifts altering 5’UTR or upstream regulatory regions and those possibly changing a protein isoform. Shifts that do not overlap with the coding region showed that the TSSs that were closer to the gene, i.e., had a shorter 5’UTR, were more frequently associated with higher expression than the more distal ones (Wilcoxon signed-rank test against zero, padj < 0.001; **Supplementary Figure S2B**). On the other hand, promoter shifts extending into the gene body were predominantly linked to decreased expression (padj < 0.01; **Supplementary Figure S2B**). Collectively, these results indicate that positional changes associated with APU substantially influence transcriptional activity.

Further, we focused on promoter-proximal regions (50 to 150 bp upstream of the TSS), examining whether promoter shifts are associated with binding of specific TFs. To identify binding motifs enriched in regions proximal to SPs compared with the global promoter-proximal background, we performed *de novo* motif discovery using STREME. This analysis revealed three significant motifs: a strong C/CT-rich motif (CCTCCCCYCCCCYCC, e-value 8.4e-15), a CG-rich motif (CGCGCGG, e-value 1.5e-4), and a less frequent GCA-repeat (GCAGCAGCAGCG, e-value 1.9e-2) (**Supplementary Table S3**). Annotation of these motifs using Tomtom revealed for the C-rich motif the strongest similarity to ERF and C2H2 binding sites, as they are predicted in PlantTFDB (barley-focused prediction). In contrast, the CG-rich motif lacked a clear match in the database, although the sequence resembles the CGCG-box *cis*-regulatory element (Yang and Poovaiah, 2002) or the binding site of LBD TFs (GCGGCG). The GCA-repeat motif shows similarity to the motif of GRF TFs in JASPAR database (Baydar Ovek et al., 2026) but lacks supporting matches in PlantTFDB. All three motifs were predominantly promoter variant-specific (**Table 1**), meaning that their occurrences were restricted to either the long or the short 5’UTR variant.

**Table 1:** Sequence motifs and two-TFBS modules enriched in shifting promoters and their promoter-variant specificity.

Enriched motif/module	Numbers of SP genes with motif/module			Proportion of variant-specific	Binomial FDR*
	present	present in both promoters	promoter variant-specific		
STREME-identified motifs					
CCTCCCCYCCCCYCC	871	252	619	0.711	1.12e-36
CGCGCGG	348	29	319	0.917	1.33e-62
GCAGCAGCAGCG	226	22	204	0.903	2.64e-38
*Cis*-regulatory modules					
ERF + ERF	112	13	99	0.884	2.63e-17
C2H2 + ERF	104	7	97	0.933	7.89e-21
ERF + MYB	46	3	43	0.935	3.24e-10
BBR-BPC + C2H2	24	3	21	0.875	1.39e-4
ERF + LBD	60	2	58	0.967	3.71e-15
C2H2 + LBD	32	5	27	0.844	6.60e-5
C2H2 + C2H2	53	3	50	0.943	4.83e-12

*relates to testing the specificity of motifs for individual promoter variants.

Finally, we examined whether the promoter-proximal regions of SPs were enriched for specific combinations of known TFBSs. Obtained TFBS hits from PlantTFDB were first merged into TF-family blocks by collapsing overlapping binding sites assigned to the same TF family. Then, local modules were defined as genomic regions containing two or more TF-family blocks located 10–50 bp apart. Fisher’s exact test identified several two-TFBS modules that were enriched in SPs (FDR < 0.05), including C2H2 + ERF, BBR-BPC + C2H2, ERF + MYB, C2H2 + LBD, and ERF + LBD (**Supplementary Table S3**). Similar to the single motifs, these enriched modules were predominantly (~90% cases) promoter variant-specific (**Table 1**). Considering the involvement of C2H2 and ERF TFs in plant responses to biotic and abiotic stresses (Rahman et al., 2025; Dey and Vlot, 2015), we assume that promoter variants containing these TFBSs could ensure a specific activation of gene expression in response to external cues.

Collectively, our results suggest that alternative promoters are involved in distinct transcriptional programs through responding to different hormonal signals or environmental stimuli. This may be the case with a gene for an achilleol B synthase-like protein from the terpene cyclase family, *HORVU.MOREX.r3.7HG0676900*. The gene exhibits two distinct TSS positions, with an internal TSS that omits the first exon. In developing and maturing embryos, both TSSs are used for transcription initiation. In the 4DAG sample, transcription is predominantly initiated from the upstream TSS (TSS1), whereas in the inflorescence, the transcription starts exclusively from the internal start site (TSS2, **Figure 2C**). Exploring sets of TFBSs in the regions proximal to both promoters revealed differences in possible upstream regulators: while MYB and ERF proteins dominate as TFs binding to the region upstream of TSS2, the region upstream of TSS1 has the highest affinity for Dof, bZIP, and AP2 TFs (**Figure 2C**). Support for the regulatory role of the APU is provided by barley pantranscriptome data (Guo et al., 2025). In barley landrace ZDM01467, TSS1 and the upstream region of the terpene cyclase gene is deleted, and the deletion is associated with a substantial reduction in the gene’s transcription in the germinating embryo, while expression remains largely unaffected in the inflorescence (**Figure 2D**). This is in line with the hypothesis that the two promoters serve non-redundant regulatory roles, presumably because each one responds to different developmental or environmental signals.

### 3.4 Promoter shifts can fine-tune translational efficiency, protein isoform and localization

Beyond the transcriptional regulation, the alternative TSS usage can reshape 5’UTR architecture and thereby influence gene expression on the post-transcriptional and translational levels. One such control element is the TOP motif. Notably, high-confidence TOP motif-associated promoters (TOPscore ≥ 3) showed largely mutually exclusive with TATA-containing promoters, with only a small fraction of SPs exhibiting both features (**Supplementary Figure S2C**). To explore whether the promoter shifts could result in regulation of gene expression via gain or loss of a TOP motif, we calculated TOPscore values for all CAGE datasets. Overall, TOP motif usage varied little across stages, with mean TOPscore difference being < 1. We identified 61 alternative transcription start cases with developmental gain or loss of a TOP motif signature (TOPscore ≥ 3 in one stage and < 2 in the other). GO term enrichment analysis of the corresponding genes highlighted processes associated with stress response, glucose signaling, and TF-related categories (**Supplementary Figure S2D**). The most contributing genes encoded TFs, such as GATA, bHLH, and heat shock proteins, as well as histidine kinases and E3 ubiquitin ligases (**Supplementary Table S4**), suggesting that TOP motif-associated shifts preferentially affect signaling and regulatory pathways.

One of the genes whose alternative promoters markedly differ in the TOPscore is *HvSUS1* (*HORVU.MOREX.r3.7HG0661420*), encoding sucrose synthase, which is a key supplier of UDP-glucose for cell-wall biosynthesis. *HvSUS1* exhibits a substantial promoter shift (1,317 bp) between early embryo and later developmental stages, associated with significant expression changes: the gene-distal TSS, dominating in the early embryo stage, shows substantially higher CAGE signal than the more proximal TSS, which becomes dominant with embryo maturation (log_2_FC > 3 for 8DAP vs. 24DAP embryo) (**Figure 3A**). Moreover, the TSS dominating in the 8DAP sample lacks the TOP motif (TOPscore ~ 0) while the alternative TSS is associated with a strong TOP motif (TOPscore ~ 13), suggesting that the translation of SUS1 could be selectively controlled by the TOR kinase pathway after the early embryonic development. Together, these findings indicate that APU could fine-tune *HvSUS1* expression at both the transcriptional and translational levels, possibly aligning the production of the sucrose synthase with changing metabolic requirements for sucrose/UDP-glucose during embryo development.

**Figure 3:**
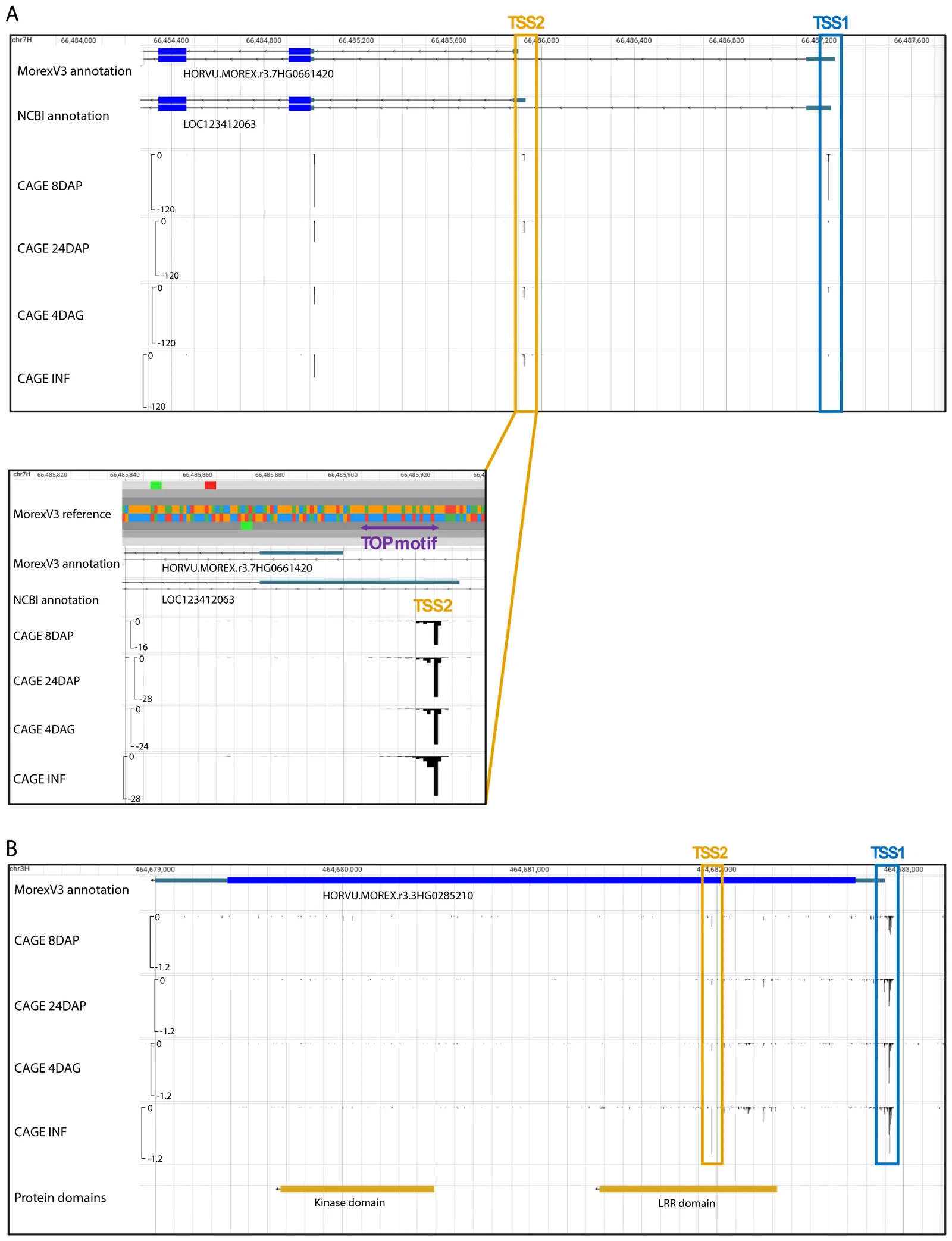
Promoter shifts can fine-tune translation efficiency through TOP motif usage and change protein isoform. **(A)** Promoter shift in the barley sucrose synthase gene *HORVU.MOREX.r3.7HG0661420* (*HvSUS1*). CAGE signal tracks show developmental switching between two transcription start sites (TSS1 and TSS2). The inset shows a zoomed view of the proximal promoter region containing a strong TOP motif associated with TSS2, while TSS1 lacks a detectable TOP motif. Note that both MorexV3 and NCBI annotations (NCBI *Hordeum vulgare* subsp. *vulgare* Annotation Release 100), which do not provide an accurate position of the TSS, would fail in revealing the TOP motif, i.e., a pyrimidine stretch starting from the TSS. Color code for the MorexV3 reference: blue - C; red - T; green - A; orange - G **(B)** A TSS shift in the barley brassinosteroid receptor gene *HORVU.MOREX.r3.3HG0285210* (*HvBRI1*). Transcription from the internal TSS2 can result in a truncated BRI1 variant with altered domain architecture. The protein domain track was generated based on data from the SMART database (Letunic and Bork, 2026).

Besides the TOP motif, the translational efficiency can be affected by the presence of uORFs in the 5’UTR. Analyzing our set of SP genes by ORFik (Tjeldnes et al., 2021), we identified 332 genes whose translational efficiency could be affected by the occurrence of uORFs in the 5’UTR due to a promoter shift (**Supplementary Table S5**).

Further, we focused on shift events involving the coding region and assessed whether these shifts could significantly alter the N-terminal region of the encoded protein and thereby affect either predicted targeting signals or domain architecture. From all genes with SPs analyzed, 155 had the alternative TSSs located within their coding regions, possibly leading to N-truncations (**Supplementary Table S6**). Using LOCALIZER (Sperschneider et al., 2017), a putative targeting peptide for the mitochondrion, chloroplast, or nucleus was predicted in 109 out of the 155 genes (**Supplementary Table S6**). Whilst for only 31 genes APU was predicted to clearly affect the presence of an N-terminal targeting signal (e.g., mitochondrial or chloroplast), another 17 genes may lose their nuclear localization signal. Because protein localization is closely linked to function, such changes can alter the cellular context, in which these proteins act. Additionally, in multi-domain proteins, promoter shifts can alter domain composition. An example is BRI1, a product of *HORVU.MOREX.r3.3HG0285210*, which is involved in brassinosteroid signaling and contains a leucine-rich repeat (LRR) and a protein kinase domains. An alternative TSS located in the exon, activated in the inflorescence sample, truncates the LRR domain (**Figure 3B**), suggesting that the resulting protein isoform may differ in functional properties.

## 4 Discussion

We used CAGE across four barley developmental stages to produce an expanded promoter atlas and to systematically characterize developmental promoter shifts. Our findings show that long-distance promoter shifts are common, affecting ~5% (1,353/25,150) of genes active in at least one of the analyzed samples, and frequently involve switches in promoter architecture and *cis*-regulon. Although approximately 20% of all active promoters contained a canonical TATA-box and about 55% matched the more permissive W-box-like definition, consistent with plant genomes (Kumari and Ware, 2013), promoter shifts occurred disproportionately in TATA-less promoters. This suggests a non-random relationship between core promoter architecture and TSS dynamics, in which TATA-less promoters may provide greater flexibility for alternative initiation (Bernardini et al., 2023). Contrary to Haberle et al. (2014), we did not observe substantial developmental variation in the relative frequency of W-box promoters across the examined stages (**Supplementary Figure S1C**). One possible explanation is that the developmental stages studied have not undergone a fundamental transition, such as the maternal-to-zygotic switch explored in zebrafish by Haberle and co-workers. Transition from vegetative to generative phase, associated with the development of inflorescence tissues, had no discernible effect on the global architecture of alternative promoters, possibly because of being limited to a small portion of cells within the inflorescence sample. To address this limitation, a targeted analysis of pollen grains or ovules would be needed.

A subset of shifts identified in this study was predicted to modify N-terminal targeting peptides or domain composition. This is consistent with previous work in Arabidopsis showing that alternative promoter selection can generate protein isoforms with different N termini and altered localization, including light-dependent relocalization of GLYK protein (Ushijima et al., 2017). Promoter shifts into the coding region can also impact domain architecture and, consequently, protein activity. In BRI1-like receptor kinase, the truncated isoform of BRI1, predicted to arise from an internal TSS, would be likely hypomorphic and could exert dominant-negative effects by interfering with productive receptor complex, similar as reported for another LRR-RLK receptor in Arabidopsis (Shpak et al., 2003). While this suggests an additional regulatory mechanism introduced by APU, we cannot exclude that the truncated protein isoforms are degraded in the secretory pathway. This uncertainty could be resolved by a comprehensive proteomic analysis, as demonstrated by Reyes et al. (2026).

A related consequence of alternative TSS choice is the remodeling of 5’UTR architecture, which may influence translational efficiency. In our previous work, we identified 558 barley genes carrying a *bona fide* 5’TOP motif signature, whose GO term enrichment analysis showed strong association with ribosomes, plastids and cell division (Pavlu et al., 2024), consistent with studies of canonical 5’TOP targets in Arabidopsis (Scarpin et al., 2020) and animals. Our current GO term analysis focused on SP genes did not point out the conventional targets of TOR signaling, suggesting that the glucose-driven control of crucial cellular processes - ribosome biogenesis and translation - does not typically involve APU. Similar to what was reported in a mammalian SP study (Philippe et al., 2020), promoter shifts in barley appear to fine-tune TOP-dependent translational control of a focused set of regulatory genes and enzymes, rather than globally reprogramming translation. It should be noted that our analysis was focused on long-distance promoter shifts, excluding biologically relevant events involving single-nucleotide or few-base changes. Since these short shifts could have a significant impact on the presence of the TOP motif and, potentially, other promoter elements, they would be worthy of a future study that would capture promoter dynamics at a fine-scale.

Changes in transcript levels, TOPscore variation and predicted changes in N-terminal protein composition suggest potential functional consequences of the APU but we acknowledge that our computational approach merely provides a prediction of its biological impact. Experimental approaches involving reporter assays to test *cis*-regulatory elements, ribosome profiling, and proteomic analysis are needed to confirm APU as a multifaceted regulatory mechanism in barley. Once validated and, in the future, extended for data from stress-affected plants, this gene-level dataset of promoter shifts provides a valuable foundation for promoter engineering aimed at fine-tuning gene expression in a tissue-, developmental stage-, or condition-specific manner, ultimately contributing to the improvement of agronomically important traits in barley.

## Data Availability

The datasets presented in this study can be found in the NBCI repository (https://www.ncbi.nlm.nih.gov) under accession numbers GSE342378 and GSE227219. The CAGE data for inflorescence are also available for visualization and download in the BarleyEpiBase (Navratilova et al., 2026) at https://olomouc.ueb.cas.cz/en/resources/barleyepibase. The scripts, high-resolution figures and other large data files (Data S1, S2 and S3) are available at https://github.com/MorexV3CAGE/Barley_CAGE_TSS_dynamics.

## References

[B1] Alfonso-Gonzalez C. Hilgers V. (2024). (Alternative) transcription start sites as regulators of RNA processing. Trends Cell Biol. 34, 1018–1028. doi: 10.1016/j.tcb.2024.02.010 38531762

[B2] Bailey T. L. (2021). STREME: accurate and versatile sequence motif discovery. Bioinformatics 37, 2834–2840. doi: 10.1093/bioinformatics/btab203 33760053 PMC8479671

[B3] Baydar Ovek D. Rauluseviciute I. Aronsen D. R. Blanc-Mathieu R. Bonthuis I. de Beukelaer H. . (2026). JASPAR 2026: expansion of transcription factor binding profiles and integration of deep learning models. Nucleic Acids Res. 54, D184–D193. doi: 10.1093/nar/gkaf1209 41325984 PMC12807658

[B4] Bernardini A. Hollinger C. Willgenss D. Müller F. Devys D. Tora L. (2023). Transcription factor IID parks and drives preinitiation complexes at sharp or broad promoters. Trends Biochem. Sci. 48, 839–848. doi: 10.1016/j.tibs.2023.07.009 37574371 PMC10529448

[B5] Carninci P. Kasukawa T. Katayama S. Gough J. Frith M. C. Maeda N. . (2005). The transcriptional landscape of the mammalian genome. Science 309, 1559–1563. doi: 10.1126/science.1112014 16141072

[B6] Cavin Périer R. Junier T. Bucher P. (1998). The eukaryotic promoter database EPD. Nucleic Acids Res. 26, 353–357. doi: 10.1093/nar/26.1.353 9399872 PMC147208

[B7] Davuluri R. V. Suzuki Y. Sugano S. Plass C. Huang T. H. (2008). The functional consequences of alternative promoter use in mammalian genomes. Trends Genet. 24, 167–177. doi: 10.1016/j.tig.2008.01.008 18329129

[B8] Dey S. Vlot A. C. (2015). Ethylene responsive factors in the orchestration of stress responses in monocotyledonous plants. Front. Plant Sci. 6, 640. doi: 10.3389/fpls.2015.00640 26379679 PMC4552142

[B9] Forrest A. R. R. Kawaji H. Rehli M. Baillie J. K. de Hoon M. J. L. FANTOM Consortium and the RIKEN PMI and CLST (DGT) . (2014). A promoter-level mammalian expression atlas. Nature 507, 462–470. doi: 10.1038/nature13182 24670764 PMC4529748

[B10] Gacita A. M. Dellefave-Castillo L. Page P. G. T. Barefield D. Y. Wasserstrom J. A. Puckelwartz M. J. . (2020). Altered enhancer and promoter usage leads to differential gene expression in the normal and failed human heart. Circ. Heart Fail. 13, e006926. doi: 10.1161/CIRCHEARTFAILURE.120.006926 32993371 PMC7577963

[B11] Grant C. E. Bailey T. L. Noble W. S. (2011). FIMO: scanning for occurrences of a given motif. Bioinformatics 27, 1017–1018. doi: 10.1093/bioinformatics/btr064 21330290 PMC3065696

[B12] Guo W. Schreiber M. Marosi V. B. Bagnaresi P. Jørgensen M. E. Braune K. B. . (2025). A barley pan-transcriptome reveals layers of genotype-dependent transcriptional complexity. Nat. Genet. 57, 441–450. doi: 10.1038/s41588-024-02069-y 39901014 PMC11821519

[B13] Gupta S. Stamatoyannopoulos J. A. Bailey T. L. Noble W. S. (2007). Quantifying similarity between motifs. Genome Biol. 8, R24. doi: 10.1186/gb-2007-8-2-r24 17324271 PMC1852410

[B14] Haberle V. Forrest A. R. R. Hayashizaki Y. Carninci P. Lenhard B. (2015). CAGEr: precise TSS data retrieval and high-resolution promoterome mining for integrative analyses. Nucleic Acids Res. 43, e51. doi: 10.1093/nar/gkv054 25653163 PMC4417143

[B15] Haberle V. Li N. Hadzhiev Y. Plessy C. Previti C. Nepal C. . (2014). Two independent transcription initiation codes overlap on vertebrate core promoters. Nature 507, 381–385. doi: 10.1038/nature12974 24531765 PMC4820030

[B16] Jayakodi M. Padmarasu S. Haberer G. Bonthala V. S. Gundlach H. Monat C. . (2020). The barley pan-genome reveals the hidden legacy of mutation breeding. Nature 588, 284–289. doi: 10.1038/s41586-020-2947-8 33239781 PMC7759462

[B17] Kovacik M. Nowicka A. Zwyrtková J. Strejčková B. Vardanega I. Esteban E. . (2024). The transcriptome landscape of developing barley seeds. Plant Cell 36, 2512–2530. doi: 10.1093/plcell/koae095 38635902 PMC11218782

[B18] Kumari S. Ware D. (2013). Genome-wide computational prediction and analysis of core promoter elements across plant monocots and dicots. PloS One 8, e79011. doi: 10.1371/journal.pone.0079011 24205361 PMC3812177

[B19] Kurihara Y. Makita Y. Kawashima M. Fujita T. Iwasaki S. Matsui M. (2018). Transcripts from downstream alternative transcription start sites evade uORF-mediated inhibition of gene expression in Arabidopsis. Proc. Natl. Acad. Sci. U.S.A. 115, 7831–7836. doi: 10.1073/pnas.1804971115 29915080 PMC6064979

[B20] Letunic I. Bork P. (2026). SMART v10: three decades of the protein domain annotation resource. Nucleic Acids Res. 54, D499–D503. doi: 10.1093/nar/gkaf1023 41062452 PMC12807790

[B21] Liu G. Y. Sabatini D. M. (2020). mTOR at the nexus of nutrition, growth, ageing and disease. Nat. Rev. Mol. Cell Biol. 21, 183–203. doi: 10.1038/s41580-019-0199-y 31937935 PMC7102936

[B22] Love M. I. Huber W. Anders S. (2014). Moderated estimation of fold change and dispersion for RNA-seq data with DESeq2. Genome Biol. 15, 550. doi: 10.1186/s13059-014-0550-8 25516281 PMC4302049

[B23] Mascher M. Gundlach H. Himmelbach A. Beier S. Twardziok S. O. Wicker T. . (2017). A chromosome conformation capture ordered sequence of the barley genome. Nature 544, 427–433. doi: 10.1038/nature22043 28447635

[B24] Mascher M. Wicker T. Jenkins J. Plott C. Lux T. Koh C. S. . (2021). Long-read sequence assembly: a technical evaluation in barley. Plant Cell 33, 1888–1906. doi: 10.1093/plcell/koab077 33710295 PMC8290290

[B25] Navratilova P. Pavlu S. Zhu Z. Tulpova Z. Kopecky O. Novak P. . (2026). Epigenome and interactome profiling uncovers principles of distal regulation in the barley genome. Cell. Genom. 6, 101037. doi: 10.1016/j.xgen.2025.101037 41075792 PMC12926201

[B26] Nepal C. Hadzhiev Y. Previti C. Haberle V. Li N. Takahashi H. . (2013). Dynamic regulation of the transcription initiation landscape at single nucleotide resolution during vertebrate embryogenesis. Genome Res. 23, 1938–1950. doi: 10.1101/gr.153692.112 24002785 PMC3814893

[B27] Pavlu S. Nikumbh S. Kovacik M. An T. Lenhard B. Simkova H. . (2024). Core promoterome of barley embryo. Comput. Struct. Biotechnol. J. 23, 264–277. doi: 10.1016/j.csbj.2023.12.003 38173877 PMC10762323

[B28] Philippe L. van den Elzen A. M. G. Watson M. J. Thoreen C. C. (2020). Global analysis of LARP1 translation targets reveals tunable and dynamic features of 5' TOP motifs. Proc. Natl. Acad. Sci. U.S.A. 117, 5319–5328. doi: 10.1073/pnas.1912864117 32094190 PMC7071917

[B29] Popp M. W. Maquat L. E. (2013). Organizing principles of mammalian nonsense-mediated mRNA decay. Annu. Rev. Genet. 47, 139–165. doi: 10.1146/annurev-genet-111212-133424 24274751 PMC4148824

[B30] Rahman M. Abid M. Abbas M. Malik W. Wei Y. Zhang R. (2025). Unraveling the Multifaceted Role of C2H2-type Zinc Finger Proteins: Integrators of trichome development and abiotic stress adaptation in plants. Plant Stress 18, 101005. doi: 10.1016/j.stress.2025.10100542574925

[B31] Reis R. S. Clúa J. Jaskolowski A. Deforges J. Jacques-Vuarambon D. Guex N. . (2024). Phosphate deficiency alters transcript isoforms via alternative transcription start sites. Plant J. 120, 218–233. doi: 10.1111/tpj.16982 39164918

[B32] Reyes A. V. Zhang C. Karunadasa S. S. Shrestha R. Grismer T. S. Byun D. . (2026). Impact of alternative splicing on Arabidopsis proteome. Nucleic Acids Res. 54, gkag400. doi: 10.1093/nar/gkag400 42080264 PMC13136898

[B33] Scarpin M. R. Leiboff S. Brunkard J. O. (2020). Parallel global profiling of plant TOR dynamics reveals a conserved role for LARP1 in translation. Elife 9, e58795. doi: 10.7554/eLife.58795 33054972 PMC7584452

[B34] Schmitz R. J. Grotewold E. Stam M. (2022). Cis-regulatory sequences in plants: Their importance, discovery, and future challenges. Plant Cell 34, 718–741. doi: 10.1093/plcell/koab281 34918159 PMC8824567

[B35] Shiraki T. Kondo S. Katayama S. Waki K. Kasukawa T. Kawaji H. . (2003). Cap analysis gene expression for high-throughput analysis of transcriptional starting point and identification of promoter usage. Proc. Natl. Acad. Sci. U.S.A. 100, 15776–15781. doi: 10.1073/pnas.2136655100 14663149 PMC307644

[B36] Shpak E. D. Lakeman M. B. Torii K. U. (2003). Dominant-negative receptor uncovers redundancy in the Arabidopsis ERECTA Leucine-rich repeat receptor-like kinase signaling pathway that regulates organ shape. Plant Cell 15, 1095–1110. doi: 10.1105/tpc.010413 12724536 PMC153719

[B37] Sperschneider J. Catanzariti A. M. DeBoer K. Petre B. Gardiner D. M. Singh K. B. . (2017). LOCALIZER: subcellular localization prediction of both plant and effector proteins in the plant cell. Sci. Rep. 7, 44598. doi: 10.1038/srep44598 28300209 PMC5353544

[B38] Supek F. Bošnjak M. Škunca N. Šmuc T. (2011). REVIGO summarizes and visualizes long lists of gene ontology terms. PloS One 6, e21800. doi: 10.1371/journal.pone.0021800 21789182 PMC3138752

[B39] Tan J. Sun Y. (2025). Alternative promoter usage during organ development. PloS Genet. 21, e1011635. doi: 10.1371/journal.pgen.1011635 40153697 PMC11978060

[B40] The ENCODE Project Consortium (2012). An integrated encyclopedia of DNA elements in the human genome. Nature 489, 57–74. doi: 10.1038/nature11247 22955616 PMC3439153

[B41] Tian F. Yang D. C. Meng Y. Q. Jin J. P. Gao G. (2020). PlantRegMap: charting functional regulatory maps in plants. Nucleic Acids Res. 48, D1104–D1113. doi: 10.1093/nar/gkz1020 31701126 PMC7145545

[B42] Tjeldnes H. Labun K. Cleuren T. Y. Chyżyńska K. Świrski M. Valen E. (2021). ORFik: a comprehensive R toolkit for the analysis of translation. BMC Bioinf. 22, 336. doi: 10.1186/s12859-021-04254-w 34147079 PMC8214792

[B43] Ushijima T. Hanada K. Gotoh E. Yamori W. Kodama Y. Tanaka H. . (2017). Light controls protein localization through phytochrome-mediated alternative promoter selection. Cell. 171, 1316–1325. doi: 10.1016/j.cell.2017.10.018 29129375

[B44] Weber R. Ghoshdastider U. Spies D. Duré C. Valdivia-Francia F. Forny M. . (2023). Monitoring the 5′UTR landscape reveals isoform switches to drive translational efficiencies in cancer. Oncogene 42, 638–650. doi: 10.1038/s41388-022-02578-2 36550360 PMC9957725

[B45] Wimalanathan K. Lawrence-Dill C. J. (2021). Gene ontology Meta Annotator for Plants (GOMAP). Plant Methods 17, 54. doi: 10.1186/s13007-021-00754-1 34034755 PMC8146647

[B46] Wu H. L. Jen J. Hsu P. Y. (2024). What, where, and how: Regulation of translation and the translational landscape in plants. Plant Cell 36, 1540–1564. doi: 10.1093/plcell/koad197 37437121 PMC11062462

[B47] Yang T. Poovaiah B. (2002). A Calmodulin-binding/CGCG Box DNA-binding Protein family involved in multiple signaling pathways in plants. J. Biol. Chem. 277, 45049–45058. doi: 10.1074/jbc.M207941200 12218065

[B48] Zhang R. Kuo R. Coulter M. Calixto C. P. G. Entizne J. C. Guo W. . (2022). A high-resolution single-molecule sequencing-based Arabidopsis transcriptome using novel methods of Iso-seq analysis. Genome Biol. 23, 149. doi: 10.1186/s13059-022-02711-0 35799267 PMC9264592

[B49] Zhang T. Wu A. Yue Y. Zhao Y. (2020). uORFs: important cis-regulatory elements in plants. Int. J. Mol. Sci. 21, 6238. doi: 10.3390/ijms21176238 32872304 PMC7503886

