# Supplementary material for: Developmental dynamics of transcription initiation highlight promoter shifts as a multifaceted regulatory mechanism in barley
Source: Front Plant Sci. 2026 Oct 29;17:1975716. doi: 10.3389/fpls.2026.1975716 (PMC13754839; doi:10.3389/fpls.2026.1975716)
Supplement: Supplementary file 1 [file DataSheet1.docx]

***Supplementary Material***

1. **Supplementary Data**

**Data S1. Consensus tag clusters identified by CAGEr across four barley developmental stages.** The clusters have been assigned to genes annotated in MorexV3.

**Data S2. Description of long-distance promoter shifts assigned to genes annotated in MorexV3.** The file includes information about differential expression and presence of TATA box and TOP motif for pairs of alternative promoters.

**Data S3.** **List of short-distance promoter shifts identified using strict criteria**

2. **Supplementary Tables**

**Table S1. Number of long and short promoter shifts identified between pairs of samples.**

**Table S2. List of SP genes with the top contribution to the GO term enrichment.**

**Table S3. Enrichment of sequence motifs and two-TFBS modules in shifting promoters versus all active promoters.**

**Table S4. List of SP genes with significant changes of the TOPscore and the top contribution to the GO term enrichment.**

**Table S5. Predicted uORFs in regions delimited by alternative TSSs.**

**Table S6. Genes with promoter shifts involving the coding region.**

3. **Supplementary Figures**

**Figure S1.** **Differences in CAGE expression profiles and characterization of promoter shifts.** **(A)** Principal Component Analysis of gene expression profiles generated from CAGE datasets across four barley developmental stages. **(B)** Relative proportions of various categories of promoter shifts, defined according to their genomic position relative to the annotated TSS (MorexV3 annotation). “Other” includes Exon & Exon, Intron & Intron, and Exon & Intron. **(C)** Proportions of TATA-box and TATA-box-less (top) and W-box and W-box-less (bottom) promoters involved in promoter shifts across four barley developmental stages.

**Figure S2. Differential expression- and TOP motif-related analyses of SP genes. (A)** Upper quartile of significantly enriched GO terms obtained for SP genes with a substantial difference in gene expression (|log2FC| ≥ 2, FDR < 0.05), shown for individual pairwise comparisons of developmental stages. The enrichment was assessed against the background of all expressed genes.  **(B)** The effect of TSS position on transcription level. Bars indicate mean log2FC in gene expression calculated (i) for a gene-proximal TSS versus a gene-distal TSS (5'UTR changes) and (ii) for a TSS within the gene body versus a TSS upstream of the gene (promoter–gene body changes). The *n* values indicate the number of shifts in each category. Statistical significance was assessed using a Wilcoxon signed-rank test against zero (** padj < 0.01, *** padj < 0.001). **(C)** Overlap between sets of shifting promoters with TATA-box and TOP motif. **(D)** Enriched GO terms for a set of genes exhibiting significant TOPscore difference between alternative TSSs. The enrichment was assessed against the background of all expressed genes.
