# Supplementary material for: Developmental dynamics of transcription initiation highlight promoter shifts as a multifaceted regulatory mechanism in barley
Source: Front Plant Sci. 2026 Oct 29;17:1975716. doi: 10.3389/fpls.2026.1975716 (PMC13754839; doi:10.3389/fpls.2026.1975716)
Supplement: Supplementary file 2 [file Table3.docx]

**Table S3. Enrichment of sequence motifs and two-TFBS modules in shifting promoters versus all active promoters.** The enrichment was calculated against a background set of all active promoters with CAGE signal > 0.1 TPM.

| **Enriched motif/module** | **Shifting promoters**  **(3073 in total)** | | **All active promoters (46643 in total)** | | **Enrichment in shifting promoters** | **Enrichment FDR*** |
| --- | --- | --- | --- | --- | --- | --- |
|  | **No. of positive promoters** | **Proportion of positive promoters** | **No. of positive promoters** | **Proportion of positive promoters** |  |  |
| **STREME-identified motifs** | | | | | | |
| CCTCCCCYCCCCYCC | 1197 | 0.390 | 8914 | 0.191 | 2.04× | 2.81e-132 |
| CGCGCGG | 394 | 0.128 | 2996 | 0.064 | 2.00× | 6.57e-35 |
| GCAGCAGCAGCG | 255 | 0.083 | 2579 | 0.055 | 1.51× | 8.41e-10 |
| ***Cis*-regulatory modules** | | | | | | |
| ERF + ERF | 129 | 0.042 | 1356 | 0.029 | 1.96× | 9.54e-9 |
| C2H2 + ERF | 113 | 0.037 | 1243 | 0.027 | 1.78× | 1.04e-5 |
| ERF + MYB | 50 | 0.016 | 414 | 0.009 | 2.23× | 2.19e-4 |
| BBR-BPC + C2H2 | 29 | 0.009 | 202 | 0.004 | 2.82× | 6.03e-4 |
| ERF + LBD | 63 | 0.021 | 696 | 0.015 | 2.20× | 0.0035 |
| C2H2 + LBD | 37 | 0.012 | 330 | 0.007 | 1.78× | 0.0035 |
| C2H2 + C2H2 | 56 | 0.018 | 648 | 0.014 | 1.72× | 0.0168 |

* Motif enrichment was assessed using Fisher’s exact test, with p-values adjusted for multiple testing using the Benjamini–Hochberg method.
